# The effect of osseodensification on implant stability and bone density: A prospective observational study

**DOI:** 10.4317/jced.56727

**Published:** 2020-05-01

**Authors:** Aseel R. Hindi, Salwan Y. Bede

**Affiliations:** 1 B.D.S. Department of Oral and Maxillofacial surgery, College of Dentistry, University of Baghdad Bab- Almoadham, Medical City, Baghdad, Iraq; 2 B.D.S., F.I.B.M.S. Professor. Department of Oral and Maxillofacial Surgery, College of Dentistry, University of Bagh­dad Bab- Almoadham, Medical City, Baghdad, Iraq

## Abstract

**Background:**

The aims of this study were to evaluate the effect of implant site preparation in low-density bone using osseodensification method in terms of implant stability changes during the osseous healing period and peri-implant bone density using CBCT.

**Material and Methods:**

This prospective observational clinical study included 24 patients who received 46 dental implants that were installed in low-density bone using the osseodensification method. CBCT was used to measure the bone density pre- and postoperatively and implant stability was measured using Periotest® immediately after implant insertion and then after 6 weeks and 12 weeks postoperatively. The data were analyzed using paired t-test and the probability value <0.05 was considered statistically significant.

**Results:**

Of the 46 implants, 43 were osseointegrated making the early survival of the implants 93.5%. There was a significant increase in bone density postoperatively; 337.6 ±182.9 compared to 265.3 ±173.9 Hounsfield units preoperatively. The primary implant stability was -2.7 ± 2.13 Periotest values (PTV), at the 6th week it decreased significantly (*p*<0.0001) to become 0.7 (± 4) PTV, and at the 12th week (secondary stability) it increased significantly (*p*<0.0001) to become -2.1 (± 2.8) PTV. The difference between primary and secondary stability was statistically non-significant (*p*=0.0814).

**Conclusions:**

Osseodensification resulted in high primary stability and increased peri-implant bone density but it did not prevent the implant stability drop during the first 6 weeks after insertion of implants.

** Key words:**Osseodensification, implant stability, low-density bone.

## Introduction

The conventional implant site preparation techniques are subtractive in nature that use successively increasing-diameter drills rotating in a clockwise direction under copious irrigation to excavate bone and prepare the implant bed (1), but recently a new non-subtractive drilling technique, osseodensification (OD), was introduced where a specially designed drills rotate in an counterclockwise direction compacting bone at the osteotomy walls allowing more intimate engagement of the implant with the osteotomy site and increasing the primary stability (2,3).

Compared with conventional drilling, OD was reported to result in higher insertion and removal torque, increased primary and secondary stability, higher bone-to-implant contact and higher bone volume around implants (4) this favorable outcome is possible because of the drills that have many lands with large negative rake angles which work as a noncutting edges to expand the implant site and increase the density of the bone (2).

After implant installation and during the osseous healing period there is a physiological drop in implant stability which accompanies the transition from primary mechanical stability to the secondary biological stability, this drop is the result of the resorption of the bone tissue immediately lateral to the implant which takes place during the initial 1-4 weeks of the healing period (5).

Despite the fact that many studies conducted on animal models have demonstrated a favorable outcome of OD over conventional drilling techniques (1,2,6-9), its clinical effect on implant stability during the osseous healing period of dental implants installed in low-density bone is not clear, therefore the aims of the study were to evaluate the effect of implant site preparation in low-density bone using OD method in terms of implant stability changes during the osseous healing period and peri-implant bone density using CBCT.

## Material and Methods

This prospective observational clinical study included 24 patients, who attended the Department of Oral and Maxillofacial Surgery at the College of Dentistry, University of Baghdad, for dental implant treatment of missing teeth, by means of delayed implant placement protocol during the period extending from December 2018 to August 2019.

The inclusion criteria were medically fit patients ≥ 18 years of age including both genders presenting with alveolar ridges of sufficient vertical and horizontal dimensions and bone density less than 850 Hounsfield units (HU) which corresponds to D3-D5 bone density according to Misch bone classification based on CBCT findings (10). Patients were excluded from the study when they had high bone density (more than 850 HU which corresponds to D1and D2), needed augmentation of the implant site, showed signs of infection in the proposed implant zone, had parafunctional habits such as bruxism and clenching, or had local limitations such as inadequate inter-ridge distance.

The institutional Research Ethics Committee approved the protocol of this study (protocol reference # 042118) and each patient signed an informed consent to participate in the study. Prior to the surgical procedure, the patients were informed about the nature of the procedure and the possible complications that may arise.

**Figure 1:**
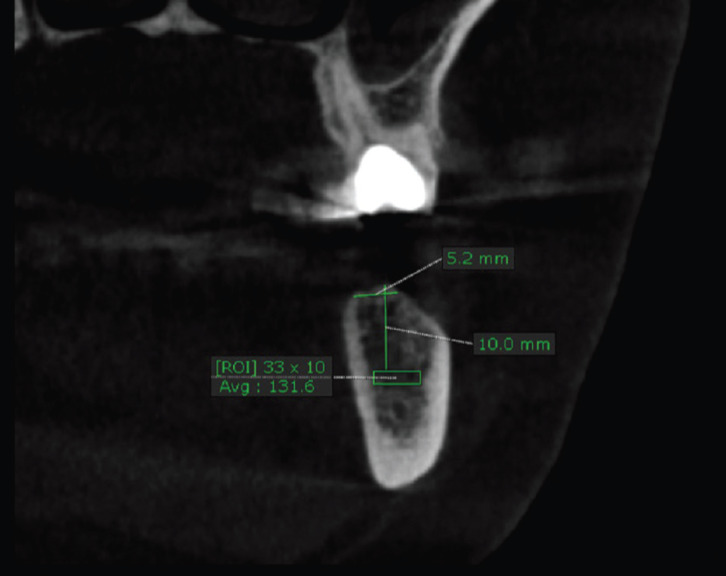
Preoperative coronal view of CBCT showing the average bone density of the area apical to the proposed implant length (131.6 Hounsfield unit).

A preoperative CBCT was taken to determine the appropriate width and length of the proposed implant and to ensure that the average bone density of the cancellous bone of the proposed area is less than 850 HU. Also in order to assess the effect of OD on bone density, a base line measurement of the bone density was recorded from the coronal view 1.5 mm apical to the proposed implant length to compare it with same area postoperatively (Fig. 1).

All the surgical procedures were performed under local anesthesia, after reflection of a mucoperiosteal flap, the implant site was prepared using Densah™ Burs (Versah Co., LLC., USA) in counterclockwise densification mode through the sequential stepped drilling. The diameter of the final drill inserted was 0.5-0.8 mm smaller than the implant diameter according to manufacturer instructions. The drilling was performed at a speed of 800 rpm and torque of 35 Ncm under copious irrigation.

The dental implants (NucleOSS™ T6, Izmir, Turkey) were installed into the osteotomy site using the motorized method with the engine set at 50 rpm and 35 Ncm torque. A ratchet was used to place the implant to the desired depth when the insertion torque required was more than 35 Ncm.

After implant placement, primary stability was measured using Periotest® M (Medizintechnik Gulden, Germany), two repeated measurements were obtained for each implant and the mean of these two readings was recorded as a Periotest value (PTV).

**Figure 2:**
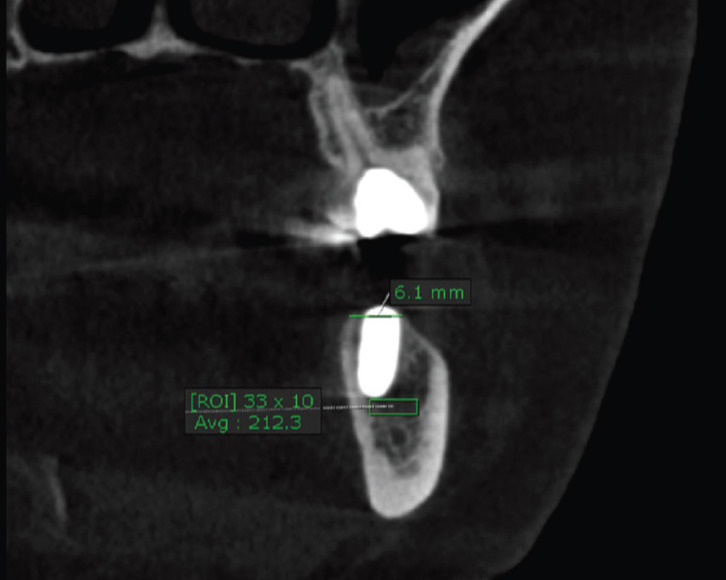
Postoperative coronal view of CBCT showing the average bone density of the same area shown in figure 1 apical to the implant length in the early postoperative period (212.3 Hounsfield unit).

A postoperative CBCT was taken within 7-10 days to measure the bone density apical to the implant within the same cross sectional view and dimensions of region of interest used preoperatively (Fig. 2). A minimum of one month was allowed between the pre- and postoperative CBCT.

Patients were instructed for follow up visits at 6 and 12 weeks postoperatively in which implant stability was measured in the same manner described in primary stability measurement. The implant stability measured at the 12th week was considered as the secondary stability. The patients were referred for final prosthesis construction after the end of the follow-up period (12 weeks).

The outcome variables in this study included the implant stability immediately after insertion (primary stability), at 6 weeks and 12 weeks (secondary stability) postoperatively to determine the pattern of implant stability changes, the bone density preoperatively and post operatively (after implant insertion) using CBCT to assess the effect of OD technique on bone density and the success rate of dental implants; success was defined as implants that were clinically sTable, pain free with no exudates after 12 weeks postoperatively (11).

The statistical analysis was performed using GraphPad Prism version 6 for Windows (GraphPad Software, La Jolla, CA, USA). Descriptive statistical analysis included calculation of percentages and mean ± standard deviation (SD) and inferential analysis included using paired t-test and the probability value <0.05 was considered statistically significant.

## Results

Twenty-four patients, 17 females (70.8%) and 7 males (29.2%) with an age range of 20-66 and a mean age (± SD) 43 (±15) years participated in this study, they received 46 implants. At the end of this study, 43 implants were osseointegrated making the early survival of the implants 93.5%.

The distribution of dental implants to the recipient jaws was equal where 23 implants (50%) were inserted in the mandible and 23 (50%) implants in the maxilla. Implants with 4.1 mm diameter were the most commonly used in this study (n=26, 56.2%), followed by implants with 3.5 mm diameter (n=20, 43.8%). With respect to the length, 10 mm implants were the most frequently used (n=21, 45.6%), followed by 12 mm implants (n=19, 41.3%) and 8 mm implants (n=6, 13.1%).

The distribution of dental implants according to bone density measurement was as follows; 18 implants (39.13%) were inserted in D4 bone (150-350 HU), 15 implants (32.61%) were inserted in D5 bone (<150 HU) and 13 implants (28.26%) were installed in D3 bone (350-850 HU).

Most of the implants (n=35, 76.1%) were inserted with an insertion torque higher than 35 Ncm, while only 11 implants (23.9%) were inserted with an insertion torque of 35 Ncm.

The mean postoperative bone density measured at the apical area of the implant site (337.6 ±182.9 HU) demonstrated a significant increase (*p* ˂ 0.0001) compared with the mean preoperative density of the same area (265.3 ±173.9 HU).

**Figure 3:**
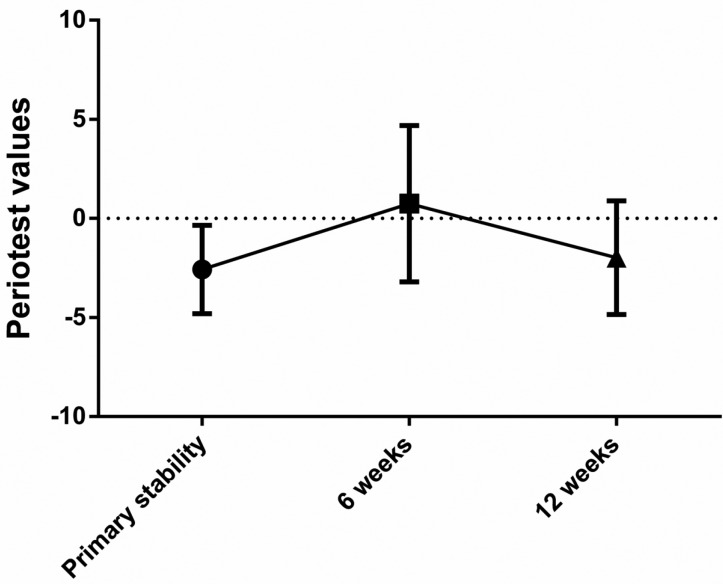
Line chart illustrating the changes in implant stability measured as periotest values (PTV) throughout the study period.

Of the 43 dental implants that were osseointegrated at the end of this study, the primary implant stability was -2.7 (± 2.13) PTV, at the 6th week it decreased significantly (*p* ˂ 0.0001) to become 0.7 (± 4) PTV, and at the 12th week (secondary stability) it increased significantly (*p* ˂ 0.0001) to become -2.1 (± 2.8) PTV. The difference between the primary and secondary stability was statistically non-significant (*p*=0.0814) (Fig. 3).

## Discussion

Achieving satisfactory primary stability in low-density bone is difficult because of the poor bone volume around the implant surface and higher rates of implant failure are reported in these cases (12-14). It is suggested that OD allows bone preservation and condensation through compaction autografting during osteotomy preparation, increasing the peri-implant bone density, and implant mechanical stability (2). The aim of this study was to investigate the effect of OD on implant stability changes throughout the healing period and to demonstrate the densification effect on bone density measured by CBCT in the early postoperative period in a low-density bone.

The primary stability achieved in this study is considered relatively high compared with that obtained after conventional drilling in low-density bone (15-17). Lahens *et al.* (6) in their animal study found that OD drilling recorded superior primary stability measured by insertion torque when compared to regular drilling irrespective of implant macrogeometry, whereas other studies demonstrated that OD did not improve primary stability (18,19).

Despite the good primary stability achieved in this study, there was a significant drop of stability during the first 6 weeks of the healing period only to increase significantly at 12 weeks compared with the stability measured at 6 weeks. This pattern of implant stability change during the healing period is also evident after implant site preparation by conventional drilling, (17,20) the drop in implant stability is associated with resorption of bone in contact with the implant surface during the first weeks of healing, the resorbed bone is replaced with newly formed viable bone which represents the transition of the implant stability from mechanical anchorage responsible for primary stability to biological attachment responsible for secondary stability (5). Contrary to this observation, some studies reported that stability either remained constant or was increased within the first 6 weeks after insertion using conventional drilling (21).

Assessment of implant stability during the osseous healing period, in this clinical study, was to determine if OD can maintain high stability levels in the early weeks after implant insertion thereby facilitating early loading, but the significant drop in stability compared with that recorded immediately after insertion of implants indicates that OD is similar to conventional drilling in this aspect, although this needs to be considered cautiously due to the lack of a control group to better assess the effect of OD on implant stability.

The majority of dental implants in this study were inserted with >35 Ncm insertion torque which is in line with the other studies (7,22). Lopez *et al.* (22) demonstrated, in an animal study, that implants installed after preparation with OD required significantly higher levels of insertion torque as compared with the regular drilling group.

In this study, the assessment of the effect of OD on bone density was confined to the apical area for 2 reasons; first, studies found that the direction of bone condensation with OD was lateral and apical to the implant body (2,23). Second, to overcome the effect of buccal and lingual/palatal cortices on the measurement of bone density of the cancellous bone (24). This study demonstrated that OD technique increased the bone density measured by CBCT apical to the implant which is supported by other histological studies (2,22,23).

The limitations of this study are mainly associated with its observational design and lack of a control group to compare the outcome variables between OD and conventional drilling, in addition to its small sample size.

## Conclusions

This study demonstrated that OD resulted in high primary stability and increased peri-implant bone density, but it did not prevent the implant stability drop during the first 6 weeks after the insertion of implants.
